# Experiences from the harmonization of Finnish national population-based health survey data

**DOI:** 10.1177/14034948211052164

**Published:** 2021-10-27

**Authors:** Laura Paalanen, Hanna Tolonen

**Affiliations:** Finnish Institute for Health and Welfare (THL), Department of Public Health and Welfare, Helsinki, Finland

**Keywords:** Health surveys, health examination survey, data harmonization, data pooling, questionnaire, self-report, biomarkers

## Abstract

*Aims:* There are several advantages to pooling survey data from individual studies over time or across different countries. Our aim is to share our experiences on harmonizing data from 13 Finnish health examination surveys covering the years 1972–2017 and to describe the challenges related to harmonizing different variable types using two questionnaire variables – blood pressure measurement and total cholesterol assessment – as examples. *Methods:* Data from Finnish national population-based health surveys were harmonized as part of the research project ‘Projections of the Burden of Disease and Disability in Finland – Health Policy Prospects’, including variables from questionnaires, objective health measurements and results from the laboratory analysis of biological samples. The process presented in the Maelstrom Research guidelines for data harmonization was followed with minor adjustments. *Results:* The harmonization of data from objective measurements and biomarkers was reasonably straightforward, but questionnaire items proved more challenging. Some questions and response options had changed during the covered time period. This concerned, for example, questionnaire items on the availability and use of medication and diet. ***Conclusions:* The long time period – 45 years – made harmonization more complicated. The survey questions or response options had changed for some topics due to changes in society. However, common core variables for topics that were especially relevant for the project, such as lifestyle factors and certain diseases or conditions, could be harmonized with sufficient comparability. For future surveys, the use of standardized survey methods and the proper documentation of data collection are recommended to facilitate harmonization.**

## Background

Pooling survey data from individual studies has several advantages, such as enhancing the statistical power of the analyses, obtaining more generalizable results, allowing cross-country comparisons and the possibility to analyze changes over time [1–3]. Commonly, projects where data are pooled have participants from several countries and even several continents. Data are often harmonized retrospectively (post-harmonization), and using common, standardized methods in data collection has not been planned in advance (pre-harmonization).

Recently, the Maelstrom Research guidelines for retrospective data harmonization were published [1]. The guidelines include a step-by-step description of a harmonization process and examples of data processing models. The steps are as follows: (0) define the research question, objectives and protocol; (1) assemble pre-existing knowledge and select studies; (2) define targeted variables and evaluate the harmonization potential; (3) process the data; (4) estimate the quality of the harmonized dataset generated; and (5) disseminate and preserve the final harmonization products. The guidelines also include essentials for successful harmonization. These include a collaborative framework, expert input, valid data input and output, rigorous documentation and respect for stakeholders. Potential pitfalls have also been listed, such as defining a realistic but scientifically acceptable level of heterogeneity or content equivalence.

Examples of large projects that have utilized the Maelstrom guidelines in their harmonization process include the Canadian Partnership for Tomorrow project [4] and the Ageing Trajectories of Health – Longitudinal Opportunities and Synergies (ATHLOS) project [5]. The step-by-step harmonization process of the Canadian Partnership for Tomorrow project data sets followed the Maelstrom guidelines closely [4]. Active interaction with individual cohorts covered the entire harmonization process and included monthly conference calls. The early phases of the harmonization process included defining the core variables to be generated. After assessing the cohort-specific data sets, a harmonization status (complete or incomplete) was assigned to each variable.

In an ongoing Finnish research project, the ‘Projections of the Burden of Disease and Disability in Finland – Health Policy Prospects’ (PoDDy-HePo), data from 13 Finnish national population-based health examination surveys from the years 1972–2017 are used [6]. Although these surveys are all from a single country, conducted by the same research organization and previously used survey protocols were considered when planning new ones, there are several challenges in the comparability of data from different surveys. Furthermore, the study objectives (listed on the project’s web page), comprise a wide range of biological and behavioral risk factors and disease outcomes (www.thl.fi/poddy-hepo). Therefore, the harmonization process was reasonably comprehensive.

## Aims

We aim to share our experiences on the harmonization process of data sets from 13 national population-based health examination surveys conducted in Finland between 1972 and 2017, and to describe challenges related to the harmonization of different variable types using four core variables as examples.

## Methods

### Harmonized surveys

**Figure 1.**
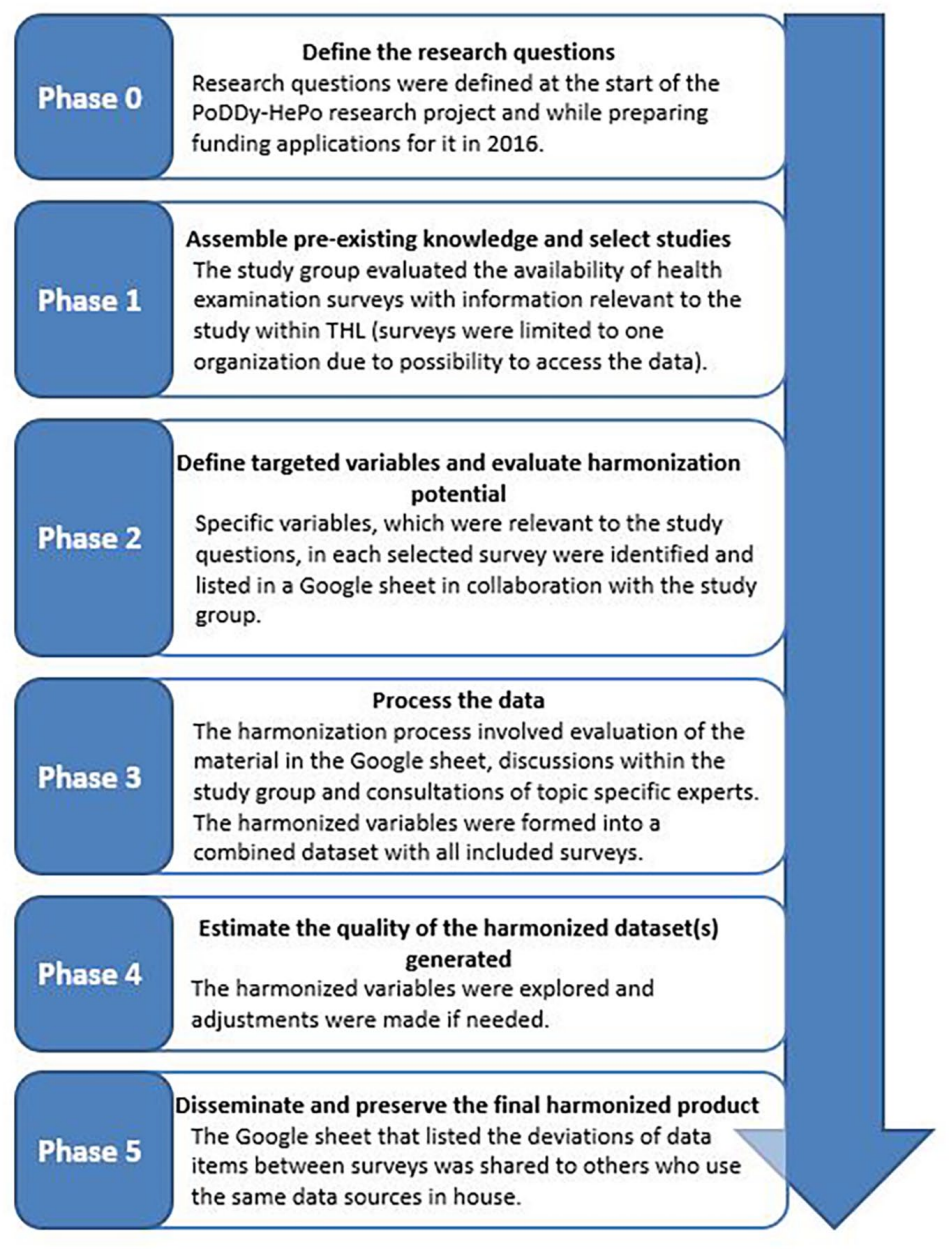
The data harmonization process of the PoDDy-HePo research project in relation to the Maelstrom Research guidelines.

The harmonization process is presented in relation to the Maelstrom Research guidelines (Figure 1).

#### Phase 0. Define the research questions

The research questions were defined by the study group as part of setting up the research project and when applying for external funding.

#### Phase 1. Assess pre-existing knowledge and select studies

The PoDDy-HePo project uses data from 13 health examination surveys carried out by the Finnish Institute for Health and Welfare (formerly the National Public Health Institute) [6]. These surveys include the FINRISK Study with a series of cross-sectional surveys conducted every five years between 1972 and 2012 [7], the Mini-Finland survey from 1978 to 1980 [8], the Health 2000 and 2011 surveys [9,10] and the FinHealth 2017 survey [11,12]. The characteristics of the participants of these surveys have been compiled in a separate protocol paper [6]. Basically, the samples were drawn from the National Population Information System to represent the general adult Finnish population or selected regions of Finland. The subjects were invited to participate with a personalized invitation letter, but additional recruitment methods were adopted in the more recent surveys, such as phone calls and SMS reminders [12]. The number of participants in the surveys ranged between 4729 and 10,938.

All surveys have followed the prevailing legislation and regulations at the time they were conducted. Written informed consent has been obtained from survey participants since 1997 but not from the participants of the early surveys that were conducted before the current legislation on medical research and the Helsinki Declaration. The participants in these early surveys were fully informed, they participated in the surveys voluntarily and the use of the data for medical and public health research was explained to them. Details on the ethical permissions of all surveys have been published previously [6].

The extent of the surveys varied somewhat. The FINRISK Study focused mainly on cardiovascular risk factors, and the Health 2000 and 2011 surveys focused more broadly on health and functional ability. The FinHealth 2017 combined the protocols from the FINRISK Study and Health 2000 and 2011 surveys but had a more limited scope than the Health 2000 and 2011 surveys. The surveys have been collected over five decades, during which, working life, family composition, diet and medical care have changed dramatically in Finland, and thus, several questions have been modified accordingly.

All surveys used questionnaires to collect data. Generally, the questionnaires were self-administered while personal interviews were carried out in part of the surveys. Basic anthropometric measurements and blood pressure measurements were performed during the health examination part of all surveys. Blood samples were drawn in all surveys, but other collected sample matrices varied between the surveys. Only biomarkers determined from blood were included in this harmonization process.

#### Phase 2. Define targeted variables and evaluate harmonization potential

Harmonization was performed only for variables relevant to the PoDDy-HePo project [6]. We started by identifying relevant variables from individual surveys. These variables included (a) self-reported data on lifestyles (smoking, diet, physical activity and alcohol consumption), background information (demographic and socio-economic variables) and selected diseases or conditions (hypertension, elevated cholesterol, diabetes, etc.); (b) data on anthropometric measurements and biomarkers; (c) information from the sampling frame such as study area; and (d) survey weights.

As an extensive list of variables was selected in our harmonization process, we focus in this paper on the description of the harmonization of variables related to one of the original aims of the project – namely, *to understand determinants of the health inequalities and their changes over time, and to assess the contribution of modifiable risk factors to these inequalities* (www.thl.fi/poddy-hepo). We present examples of the harmonization of the following variables, which were essential in analyses related to this aim:

- the fat type used in cooking;
- a question on the use of cholesterol-lowering medication;
- blood pressure measurement;
- assessment of total cholesterol.

### Platform for variable harmonization

#### Phase 3. Process data

To facilitate the harmonization process, we created a Google sheet platform in which we inserted information for those variables from all surveys that we aimed to harmonize. The individual surveys were organized in columns, whereas the variables were inserted in rows.

The first column served as a title column with a general description of each variable. In the following columns, information available from each specific survey for this variable was filled in.

**Figure 2.**
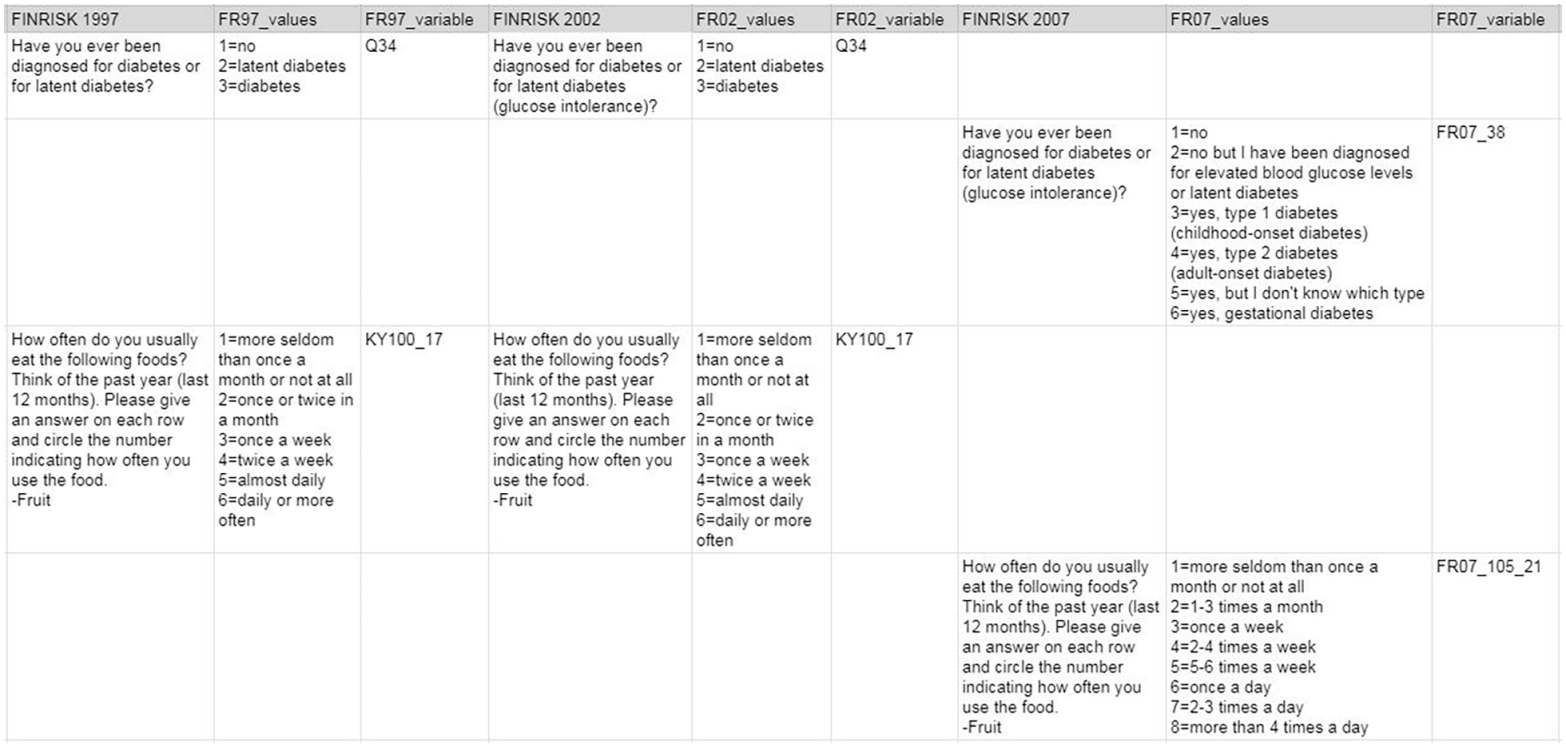
Examples of two questionnaire items from three surveys as inserted into the Google sheet platform.

Each survey took up three columns. The columns for variables based on survey questionnaires included the following information: (1) the exact wording of the original question, (2) the response categories (e.g. 1 = yes, 2 = no) and (3) the variable name in the original data set (Figure 2).

The content of columns for variables based on measurements and for biomarkers determined from biological samples was modified. For these variable types, the content of the columns was basically as follows: (1) description of the measurement or biomarker, (2) the values for categorical variable / the unit for continuous variables (e.g. mmol/l) and (3) the variable name in the original data set.

Fully comparable variables across individual surveys were written on the same row. If there was a deviation in the formulation of the question/response options, measurements or biomarkers, information was inserted into a new row. This arrangement of variables helped the inspection of the variables and their differences during later phases of the project.

Before the final selection of core variables to be used in the project, the study group discussed harmonization alternatives for the included variables, such as the number and content of the categories for different categorical variables. When there were doubts about the feasibility of the proposed categorization, the frequencies were checked and adjusted. Topic-specific experts were consulted, either in person or by email, to check the categorization of response options and the cut-offs of continuous variables regarding alcohol-related variables, for example. Based on these expert consultations and discussions among the study group in project meetings, the final selection of the harmonized core variables was decided on. Issues that were considered in the final selection were mostly related to possibilities to form comparable variables. For example, the time span that a specific question referred to varied between the surveys for some variables, which hindered comparability. No formal criteria for the final variables were used, but each variable was discussed separately.

#### Phase 4. Estimate the quality of the harmonized dataset generated

Finally, the harmonized variables for each survey were created using R following the coding instructions documented in the codebook, and one combined dataset including all surveys was formed. The harmonized variables were given common variable names.

#### Phase 5. Disseminate and preserve the final harmonized product

The Google sheet, including the documentation and codebook for variable harmonization, was made available for other in-house users of the same data sources.

As some of the research questions took shape gradually during the project, the process was repeated later for other required variables.

## Results

Generally, the prerequisites for harmonization of variables from more recent surveys were reasonable. However, the questions from the surveys conducted during the 1970s differed from more recent surveys.

### Challenges in the comparability of the data

#### Questionnaire-based data

The questionnaires used in the individual surveys mainly included multiple-choice questions and only some open-ended questions. Harmonization of many of these questions was reasonably straightforward. For many questions, the wording of the question itself was identical across the surveys, but the response options varied. In most of these cases, it was generally feasible to form a harmonized variable by combining the response categories.

The question on the type of fat used in cooking is a good example of the changes in the response categories of multiple-choice questions over the course of time. This specific question has changed over time because the range of foods available in grocery stores has grown. In 1972, the FINRISK questionnaire included four response categories for the question on the type of fat used in cooking, whereas 40 years later (in 2012), the number of categories was nine (Table I). Information on the more thorough classification of the more recent studies was inevitably lost during harmonization when categories that would be possibly comparable with earlier categories were formed. One of the harmonized variables generated from this question was a dichotomous variable: 1 = *vegetable oil or other mainly unsaturated fat*; 0 = *all other options* (presented in Table I).

**Table I.** Harmonization of variable ‘fat used in cooking’ from specific questions used in FINRISK surveys in 1972–2012, Health 2000/2011 surveys and FinHealth 2017 survey.

	A	B	C	D	E	F	G
Surveys where this format was used	FINRISK 1972	FINRISK 1977	FINRISK 1982 and FINRISK 1987	FINRISK 1992	FINRISK 1997, FINRISK 2002 and Health 2000	FINRISK 2007, FINRISK 2012 and FinHealth 2017	Health 2011
Response options for question: *What kind of fat is usually used for cooking in your household?*	1) We don’t cook in our household 2) Vegetable oil 3) Hard margarine 4) Butter	1) We don’t cook in our household 2) Vegetable oil 3) Soft margarine 4) Regular margarine 5) So-called soft butter 6) Butter	1) Vegetable oil 2) Soft margarine 3) Regular margarine 4) Mixture of butter and vegetable oil 5) Butter 6) We don’t cook in our household	1) Vegetable oil 2) Margarine spread with 60% fat 3) Margarine spread with 70–80% fat 4) Hard margarine 5) Mixture of butter and vegetable oil 6) Butter 7) No fat used in cooking	1) Vegetable oil 2) Margarine spread with 60% fat 3) Margarine spread with 70–80% fat 4) Cooking margarine 5) Butter–vegetable oil mixture 6) Butter 7) Vegetable sterol margarine 8) No fat at all	1) Vegetable oil or liquid margarine 2) Fat spread with 60% fat 3) Fat spread with 70–80% fat 4) Hard margarine 5) Mixture of butter and vegetable oil 6) Butter 7) Plant sterol margarine 8) No fat at all 9) We don’t cook in our household	1) Vegetable oil 2) Fluid vegetable oil product or fat mixture 3) Spread with about 60% fat 4) Margarine or fat spread with 70–80% fat 5) Household margarine 6) Mixture of butter and vegetable oil 7) Butter 8) No fat at all
Harmonized variable: 1 = vegetable oil or other mainly unsaturated fat 0 = other fat types, no fat used in cooking or no cooking in the household (Response options classified into the categories of the harmonized variable are listed after the arrow)	1 ← 2 0 ← 1, 3, 4	1 ← 2, 3 0 ← 1, 4–6	1 ← 1, 2 0 ← 3–6	1 ← 1–3 0 ← 4–7	1 ← 1–3, 7 0 ← 4–6, 8	1 ← 1–3, 7 0 ← 4–6, 8–9	1 ← 1–4 0 ← 5–8

New drugs over the five decades also resulted in changes in the survey questions. For example, the first commercial statin for lowering cholesterol levels was approved by the US Food and Drug Administration in 1987 [13]. Thus, a question on the use of cholesterol-lowering medication was added to the FINRISK questionnaire in 1992. One of the selected core variables in the current project was elevated total cholesterol. The variable was formed as a combination of measured and self-reported information, with elevated total cholesterol defined as measured serum total cholesterol levels ⩾5.0 mmol/l and/or self-reported use of cholesterol-lowering medication. However, for the studies from the 1970s and 1980s, from the time when cholesterol-lowering medication was not yet available and not included in the questionnaire, the harmonized variable was created on the basis of measured serum total cholesterol only.

Moreover, the treatment guidelines for some conditions have changed. Questions like, ‘Have you ever been diagnosed for high blood cholesterol level?’ are not directly comparable if the threshold for high cholesterol has changed. Our harmonized surveys cover 45 years, and the thresholds for elevated cholesterol and elevated blood pressure have changed. Therefore, we used measured cholesterol and blood pressure values together with information on the use of medication as the primary information sources for these conditions.

#### Measurements performed during a health examination

The anthropometric and blood pressure measurements have been performed by trained study nurses in all included surveys. The measurement techniques followed standardized protocols, which usually remained unchanged between surveys, and there were only a few issues to consider in the harmonization process.

Blood pressure has been measured with a sphygmomanometer in all surveys following the methods originally published by Rose and Blackburn in 1968 [14], and later in the WHO MONICA Project protocol [15] and the *EHES manual* [16]. The measurement protocol has not changed, but the number of blood pressure measurements has varied. In the first FINRISK survey in 1972, blood pressure was measured only once. Five years later, in 1977, a second measurement was added to the study protocol. Since 2002, blood pressure has been measured three times in FINRISK surveys, and this practice continued in the FinHealth 2017 survey. In the Health 2000 and 2011 surveys, blood pressure was measured twice. For the harmonized data, we calculated the means for systolic and diastolic blood pressure values using the first and second measurements and used the first measurement only for the FINRISK 1972 survey.

#### Laboratory samples and analyses

All laboratory analyses have been performed at the same laboratory – that is, the laboratory of the Finnish Institute for Health and Welfare (formerly the National Public Health Institute), except for the first FINRISK survey in 1972. The analytical methods have changed for some biomarkers. However, the laboratory of the Finnish Institute for Health and Welfare has taken part in the quality control programs of the WHO MONICA Quality Control Centre for Lipid Measurements in Prague between 1978 and 1997 and the Centers for Disease Control and Prevention, Atlanta, USA, since 2002 [7], which has enabled an accurate estimation of systematic error for each survey. As the systematic error for serum total cholesterol has been examined based on external quality control, we were able to correct the values with the respective coefficients for each survey [17,18].

## Discussion

We described the harmonization process of 13 Finnish health examination surveys covering 45 years between 1972 and 2017, using four core variables as examples. Our harmonized variables included data collected by questionnaires, objectively measured data and biomarkers from laboratory analyses. Compared to research projects where data from several countries were collected and harmonized, our harmonization process was probably more straightforward. The same research institute has been responsible for data collection of all included surveys and has aimed to keep survey protocols as similar as possible. Nonetheless, the harmonization process took a considerable amount of work, consultations, meetings and discussions in the study group. It imitated the steps of the Maelstrom Research guidelines, where the harmonization process proceeds from the first step of defining the research question, objectives and protocol to the final step of disseminating and preserving the final harmonization products [1].

One of the strengths in our harmonization process was that substantial consideration and effort had been put into the standard operating procedures of the surveys in the planning phase as well as in thorough training of the survey nurses. Core variables, such as measured blood pressure, did not need rigorous harmonization. Furthermore, the laboratory methods and their changes have been extensively reported previously and the systematic errors between the measurements have been assessed [17–19], which made the harmonization of the core biomarkers such as total cholesterol easier.

By contrast, some of the variables that were obtained from questionnaires were more challenging to harmonize. In many cases, when the wording of the question itself was identical across the surveys, it was possible to combine the response categories into common target categories. Revising the wording of a question or its response categories in new survey rounds may, however, lead to variables that are not feasible to harmonize. For continuous variables, this hindrance does not exist as the cut-off points may be set flexibly.

Cross-country harmonization may be especially challenging if cultural differences are reflected in different questions or response options. This can be seen, for example, in diet-related questions, which commonly include foods that are common in the food culture of a country as well as locally available. This was seen in the BioSHaRE project, where data from six European countries were pooled. Dietary habit variables clearly showed lower harmonization potential than disease history and medication use, as examples [20]. In our project, all harmonized surveys came from Finland, but the surveys covered 45 years and the dietary habits and availability of food products have changed considerably over time, and response categories for some food groups were increased. Quantitative data on food consumption and nutrient intake measured with a quantitative food frequency questionnaire might be more straightforward to harmonize than more general questions on dietary habits.

Overall, using standardized protocols such as those developed in the framework of the European Health Examination Survey (EHES) [16,21] facilitates research collaboration and enables the comparability of data without the need for excessive efforts and possible compromises during the data harmonization process.

It should be kept in mind that pre-harmonization (i.e. the use of standardized survey methods and protocols for data collection) is always easier than post-harmonization of already collected data. Obviously, the primary use of the collected data will guide the decisions on the data collection methods, especially when a new survey is a continuation of a series of earlier surveys. We often have to balance between two choices: (a) comparability with earlier surveys and thereby the possibility for trend analyses, or possibly even comparability with corresponding international surveys; and (b) the possibility to obtain accurate and relevant data using updated, nationally relevant, topical and/or validated survey questions and measurement protocols. Survey data collected for one purpose (e.g. the monitoring of the health status and health determinants of the general population) are often also used for secondary purposes, such as research collaborations. In these cases, we usually must content ourselves with previously collected data and work on post-harmonization of the data. It is also essential to have proper documentation of the data collection available to support the harmonization process. Information about societal changes, development of the used treatments and treatment guidelines may affect the interpretation of the questions/measurements and should be used to guide the harmonization process.

## Conclusions

The harmonization of variables, which are based on objective measurements performed by trained study nurses in the health examination part of a survey as well as biomarkers measured from blood, were fairly straightforward to harmonize. The harmonization of questionnaire-based variables was more challenging. The surveys in the current harmonization process covered 45 years, and the questions or response options had changed for some topics, such as dietary habits. New questions on, for example, the use of medications had also been added. This provided an additional layer of complexity to the harmonization process. Common core variables for topics that were especially relevant for the project, such as lifestyle factors and certain diseases or conditions, could be harmonized with sufficient comparability.

In general, pre-harmonization – that is, the use of standardized survey methods and protocols for data collection – is easier than post-harmonization of already collected data. Whenever possible, this is worth acknowledging in connection with future data collections. Proper documentation of the data collection also facilitates data harmonization in both pre- and post-harmonization processes.
